# Associations between proximity to livestock farms, primary health care visits and self-reported symptoms

**DOI:** 10.1186/s12875-016-0421-3

**Published:** 2016-02-19

**Authors:** Christel E. van Dijk, Lidwien A. M. Smit, Mariette Hooiveld, Jan-Paul Zock, Inge M. Wouters, Dick J. J. Heederik, C. Joris Yzermans

**Affiliations:** NIVEL, Netherlands Institute for Health Services Research, Utrecht, The Netherlands; Utrecht University, Institute for Risk Assessment Sciences, Division Environmental Epidemiology, Utrecht, The Netherlands; Centre for Research in Environmental Epidemiology (CREAL), Barcelona, Spain; Universitat Pompeu Fabra (UPF), Barcelona, Spain; CIBER Epidemiología y Salud Pública (CIBERESP), Madrid, Madrid Spain

**Keywords:** Environmental exposure, Respiratory system, Delivery of health care, Primary health care, Livestock

## Abstract

**Background:**

Living in a neighbourhood with a high density of livestock farms has been associated with adverse respiratory health effects, but less is known about healthcare utilisation. This study aimed at investigating the associations between livestock exposure and primary health care visits and self-reported symptoms. In addition, we examined the potentially confounding effect of distance from home to general practice.

**Methods:**

Contact data between 2006 and 2009 were obtained from electronic medical records of 54,777 persons registered within 16 general practices in an area with a high density of livestock farms in the Netherlands. Data on self-reported symptoms were used from a cross-sectional sample of 531 patients in 2010. Livestock presence in a 500 m radius from home was computed using Geographic Information System data.

**Results:**

In general, livestock exposure was associated with fewer contacts and self-reported symptoms for respiratory and other conditions. The number of poultry within 500 m was positively associated with the number of contacts. A longer distance to general practice was associated with fewer contacts, but did not confound associations.

**Conclusions:**

People living close to livestock farms less often see their general practitioner and report symptoms.

## Background

Living in a neighbourhood with a high density of livestock farms has been associated with adverse respiratory health effects [1–3]. Livestock farms are known to contain several compounds, including microbial compounds such as bacteria, endotoxins, fungi, viruses, pathogenic infectious agents, and also particular matter (PM), ammonia, hydrogen sulphide (H_2_S) and volatile organic compounds [4]. Through ventilation and diffusion, these compounds may emit to the environment. However, information on exposure levels in the vicinity of livestock farms in general is scarce [4]. Several studies showed a higher prevalence of wheezing and difficulty in breathing, and lower lung function with increased livestock exposures in residents near farms [1–3]. However, the association between livestock exposures and the prevalence of respiratory diseases is inconclusive. One study found a lower prevalence of asthma, COPD and allergic rhinitis, one study a higher prevalence of asthma and two studies found no difference in the prevalence of asthma or allergic rhinitis with increased livestock exposures [1, 3, 5, 6]. The association between exposure to livestock and healthcare utilisation was not examined until now.

As areas with a high density of livestock farms are located in rural areas with a lower geographical density of general practices, access to healthcare might be compromised compared to more urban areas as travel time to healthcare providers is increased. Literature with regard to travel time or distance to healthcare and healthcare utilisation points towards decreased healthcare utilisation with increased distance [7]. Thus, higher respiratory healthcare utilisation due to respiratory health problems with increased livestock exposure may be counterbalanced by lower accessibility of healthcare. In other words, distance to healthcare providers might be an important confounder in the association between exposure to livestock exposures and primary healthcare utilisation.

The objective of this study is to illuminate the association between livestock exposure and healthcare utilisation, in terms of contacts in general practice, using data from electronic medical records (EMRs) of general practices of residents living in an area in the Netherlands with a high density of livestock farms. This study addresses the following questions:

*What is the association between livestock exposure and (respiratory) contacts in general practice? Does distance to general practice confound associations between livestock exposure and (respiratory) contacts?*

Lower healthcare utilisation could both indicate a lower demand for healthcare and a lower accessibility of healthcare. To be fully certain that possible differences (after adjusting for distance to general practice) represent differences in demand and not accessibility, we additionally analysed self-reported symptoms of a subgroup of patients. The self-reported symptoms can be seen as an indicator for latent demand.

## Methods

### Study design and population

Data collection was described previously [5]. In short, general practices outside the larger cities in the south-eastern part of the Netherlands were requested to participate. Fifty-five practices agreed to participate, of which 27 met pre-defined registration quality criteria (e.g. full yearly registration of morbidity) regarding their EMR data. For the present study, additional quality criteria with regard to general practice contacts (e.g. year-round registration of contacts) were applied as this was not part of the pre-defined registration criteria. This led to the inclusion of 16 practices with contact data between 2006 and 2009. As we are interested in people living close to livestock farms and not people living on farms (as the exposure level of people living on farms is much higher and at levels known to have adverse respiratory health effects, which could confound associations), persons with a high likelihood of living on a farm were excluded (distance between home address and livestock farms <50 m – see paragraph livestock exposure; *n* = 2,374).

In total, contact data in general practice of 54,777 persons and 144,984 person years were included: on average 2.65 years of follow up per person. Reason for contacts were recorded in the EMR of general practices using the International Classification of Primary Care (ICPC [8]). All Dutch inhabitants are obligatory listed in a general practice and the general practitioner (GP) acts as gatekeeper for specialized, secondary healthcare. Therefore, the EMR kept by the GP provides the most complete picture of people’s health. The respiratory health of the study population is similar to the general population in the Netherlands [9]. For example the prevalence of acute upper respiratory tract infections and influenza is respectively 66.7 and 8.4 per 1000 patients in the study population and 69.6 and 8.6 per 1000 patients in the general population (data 2013).

To analyse the association between self-reported symptoms and livestock exposures, secondary analyses were performed on a cross-sectional sample of 1,519 patients diagnosed with lower back pain without radiation in 2009, who were randomly selected from the adult GP patient population (≥18 years) of 20 general practices participating in the study (same population as data on contacts). These data were part of a case–control questionnaire study analysing the potential confounding in the association between livestock exposure and asthma [10]. As patients were diagnosed with lower back pain in 2009, these patients did not necessary attended primary health care in 2010. Patient with lower back pain were selected as lower back pain is common in all ages, having lower back pain is believed to be independent of environmental livestock exposure (comparison between rural areas with high and low livestock density show similar prevalence rates: 77.3 versus 78.4 per 1000 registered patients (OR: 0.98 95 % CI: 0.93–1.04)), and because having lower back pain is not causally related to respiratory symptoms. Therefore, we were able to analyse the association between self-reported symptoms and livestock exposures in this sub population as it was almost identical to the general population. In June 2010, patients received a questionnaire via their GP addressing among others self-reported symptoms. In total, 662 patients returned a completed questionnaire (response 44 %) of which 531 had no missing data. Analyses of non-responders showed that participants were more often female, of higher age and the distance to AFO was smaller compared with non-participants [10]. Previous research showed that only a small percentage of the patients, around 5 %, visited their GP for their symptoms, indicating that most self-reported symptoms are not severe enough to require a visit to their GP [11].

Privacy was ensured by keeping medical information and address records separated at all times, by using a Trusted Third Party. According to the Dutch Medical Research Involving Human Subjects Act this study did not require ethical approval.

### Livestock exposures

Data on farm characteristics in the study area (geographic location, type and number of animals) were obtained from the provincial database of mandatory environmental licences for keeping livestock in 2009. Participants’ residential addresses were geocoded, and distances between the home address and all livestock farms within a 500 m radius were calculated using a geographic information system (ArcGis 9.3.1, Esri, Redlands, CA). A distance of 500 m was chosen as a previous study showed differences in respiratory health in subjects living within 500 m of a livestock farm [1]. The following livestock exposure variables were considered: 1) distance to the nearest farm; 2) presence of one or more farms within 500 m from the home address; 3) total number of farms within 500 m; 4) presence and number of specific farm animals (swine, poultry, cattle, goats, sheep, or minks) within 500 m; and 5) inverse-distance weighted Particulate Matter up to 10 micrometers in size (PM10). PM10 emission from all farms within 500 m (see for detailed description modeling PM10 emission Appendix 1). Livestock farming is a major source of PM air pollution in the study area, and measured PM10 and endotoxin were positively associated with the number of farms around sampling locations [12]. The livestock exposure variables related to the specific farm animals were only weakly correlated.

### Distance to general practice

Euclidian distance in meters was calculated between the home address and the general practice address. As the correlation between travel time by car and Euclidian distance might differ within rural areas, we calculated the travel time by car for a subset of 120 randomly selected study subjects using a publicly available widely used route planner. These analyses showed a strong correlation between travel time by car and Euclidian distance below 4 km (r = 0.85, *p* < 0.0001), but low correlations above 4 km (r = -0.18, *p* = 0.62). For this reason, sensitivity analyses were conducted by excluding study subjects living at more than 4 km of their general practice (*n* = 17,054; 11.8 %).

### Contacts with general practice for respiratory conditions

Contacts with the general practice were based on claim data (GPs are reimbursed for every contact they claim at the health insurance company). Contacts included consultations (68 %), home visits (5 %) and telephone consultations (27 %). We considered all contacts and specific contacts for respiratory symptoms, respiratory illnesses and acute respiratory infections.

### Respiratory tract symptoms

The subpopulation of patients with lower back pain was asked to report whether they experienced any of the listed 26 different symptoms of which four respiratory (cold/flu, cough, shortness of breath/ difficulty breathing and sore throat) in the last month. Both the total number of self-reported symptoms and the total number of self-reported respiratory symptoms were included as health outcome variables in the analyses.

### Statistical analyses

Associations between the different livestock exposure variables and contacts (dependent variable) were analysed with mutually adjusted multilevel Poisson regression analyses, using a model with three-levels, since the data are hierarchically structured (persons years nested within persons and persons nested within general practices). Practice variation on the intercept was estimated for each year separately and on person level for all years together. In the first model, associations were adjusted for age, gender and the number of chronic diseases (list of chronic diseases used by van Oostrom et al. [13]), and in the second model for distance to general practice also. The association between livestock exposures and the number of self-reported symptoms (dependent variable) was analysed with multiple Poisson regression analyses (multilevel analyses showed similar results, data not shown) with correction for overdispersion (variance larger than the mean). In the first model, associations were adjusted for age and gender, and in the second model for distance to general practice as well. Number of animals were categorized in equal groups plus a ‘no animal’ category as the reference category. Weighted PM10 emissions, distance to the nearest farm and distance to general practice were log-transformed to reduce skewness. Relative Risks (RR) and 95 % confidence intervals (CI) associated with a change in exposure over the interquartile range (IQR) increase were calculated for transformed exposure measures. Analyses were performed using Stata 12 and MLwiN 2.30.

## Results

### Characteristics of the study population

Characteristics of the study population are shown in Table 1. The geometric mean distance to the nearest farm was 351 m and the geometric mean distance to general practice 970 m. More than half of the participants (53 %) had a farm with cattle and 39 % had a swine farm within 500 m of their home address. Proximity within 500 m of poultry and sheep was less common (15 % and 13 %, respectively). On average people had 3.77 contacts per year with their general practice, of which 0.15 contacts for respiratory symptoms and 0.32 contacts per year for respiratory diagnoses (0.45 in total). The contact rate is similar to the general population in the Netherlands (total contact rate 3.91 per year and contact for respiratory symptoms and diagnoses 0.49 per year; [14]).

**Table 1:** Characteristics of the study population, 2006–2009

Characteristic	
Person years, n (%)	144,984
Female gender, n (%)	71,391 (49.2)
Age, years (mean (SD))	39.7 (22.2)
Chronic diseases, nr (mean (SD))	0.31 (0.64)
Distance to general practice, m (GM (IQR))	970 (470–2330)
Contacts, nr (mean (SD))	3.77 (4.83)
Contacts for respiratory symptoms, nr (mean (SD))	0.15 (0.61)
Contacts for respiratory diagnoses, nr (mean (SD))	0.32 (1.07)
Contacts for acute respiratory infections, nr (mean (SD))	0.14 (0.60)
Distance weighted PM10 emission from farms within 500 m, g∙y^-1^∙m^-2^ (GM (IQR))	0.0437 (0.0001–3.0000)
Distance to the nearest livestock farm, m (GM (IQR))	351 (250–570)
One or more farms within 500 m, n (%)	95,074 (65.6)
Livestock farms within 500 m, nr (mean (SD))	1.94 (2.23)
Presence of farm animals within 500 m, n (%)	
Cattle	
1–69	22,707 (15.7)
70–299	26,902 (18.6)
300–4,210	26,786 (18.5)
Swine	
1–649	18,891 (13.0)
650–1,999	17,737 (12.2)
2,000–32,660	20,157 (13.9)
Poultry	
1–13,999	10,848 (7.5)
14,000–401,250	10,731 (7.4)
Sheep	
1–49	10,529 (7.3)
50–1,400	8,324 (5.7)
Mink	5,834 (4.0)
Goat	4,291 (3.0)

*GM* geometric mean, *IQR* interquartile range

### Associations between livestock exposures and contacts in general practice

Increased distance from home to general practice was associated with a lower number of contacts (Table 2). Most livestock exposure measures were inversely associated with the total number of contacts and with the number of contacts for respiratory symptoms, respiratory diagnoses and acute respiratory infections. Distance to the nearest farm, weighted PM10 emission and number of farms within 500 m all showed a lower contact rate with increased livestock exposure. The presence and number of specific livestock (cattle, swine, poultry sheep, minks and goats) within 500 m was not always associated with a lower contact rate. Of interest is the positive association between the highest category of poultry exposure within 500 m and contacts in general practice (RR: 1.04; 95 % CI:1.01–1.07), contacts for respiratory diagnoses (RR: 1.09; 95 % CI:1.00–1.18) and for acute respiratory infections (RR:1.17; 95 % CI:1.06–1.29). Distance to general practice did not show to be a confounder in these associations (Appendix 2). Sensitivity analyses including only study subjects living within 4 km of their general practice showed similar associations (not shown).

**Table 2:** Association of livestock exposures and contacts in general practice in 144,984 person years between 2006 and 2009

	All contacts	Contacts for respiratory symptoms	Contacts for respiratory diagnoses	Contacts for acute respiratory infections
	RR (95 % CI)	RR (95 % CI)	RR (95 % CI)	RR (95 % CI)
Distance to general practice^a^	**0.94 (0.93**–**0.95)**	**0.94 (0.91**–**0.98)**	**0.96 (0.94**–**0.99)**	**0.95 (0.92**–**0.99)**
*Livestock exposures*				
Distance weighted PM10 emission from farms within 500 m ^a^	**0.94 (0.92**–**0.95)**	**0.87 (0.83**–**0.92)**	**0.92 (0.88**–**0.96)**	**0.93 (0.88**–**0.98)**
Distance to the nearest farm ^a^	**1.05 (1.04**–**1.06)**	**1.10(1.06**–**1.14)**	**1.07 (1.04**–**1.10)**	**1.08 (1.04**–**1.11)**
One or more farms within 500 m (ref no farms)	**0.95 (0.93**–**0.97)**	**0.90 (0.86**–**0.95)**	**0.93 (0.89**–**0.97)**	**0.93 (0.88**–**0.98)**
Number of farms within 500 m	**0.98 (0.98**–**0.99)**	**0.97 (0.96**–**0.98)**	**0.98 (0.97**–**0.99)**	**0.98 (0.97**–**1.00)**
Presence of farm animals within 500 m				
Cattle (ref no cattle)				
1–69	0.99 (0.97–1.02)	1.00 (0.92–1.08)	0.98 (0.92–1.05)	0.98 (0.90–1.06)
70–299	**0.95 (0.93**–**0.97)**	0.98 (0.91–1.06)	0.95 (0.89–1.01)	0.95 (0.88–1.03)
300–4,210	**0.94 (0.92**–**0.97)**	0.94 (0.87–1.02)	0.95 (0.88–1.01)	0.94 (0.86–1.02)
Swine (ref no swine)				
1–649	1.01 (0.99–1.04)	**0.87 (0.80**–**0.95)**	0.99 (0.92–1.07)	1.02 (0.94–1.11)
650–1,999	0.98 (0.96–1.01)	0.92 (0.85–1.00)	0.95 (0.88–1.01)	0.99 (0.91–1.07)
2,000–32,660	**0.93 (0.91**–**0.95)**	**0.82 (0.76**–**0.90)**	**0.89 (0.83**–**0.95)**	**0.88 (0.81**–**0.96)**
Poultry (ref no poultry)				
1–13,999	0.98 (0.95–1.01)	**0.89 (0.80**–**0.99)**	1.03 (0.95–1.12)	0.99 (0.89–1.10)
14,000–401,250	**1.04 (1.01**–**1.07)**	1.07 (0.97–1.18)	**1.09 (1.00**–**1.18)**	**1.17 (1.06**–**1.29)**
Sheep (ref no sheep)				
1–49	0.99 (0.96–1.02)	1.03 (0.92–1.14)	1.01 (0.93–1.10)	0.98 (0.88–1.09)
50–1400	1.00 (0.96–1.03)	1.04 (0.93–1.16)	1.02 (0.93–1.12)	1.00 (0.89–1.12)
Minks (ref no minks)	**0.93 (0.89**–**0.97)**	0.92 (0.80–1.05)	0.97 (0.86–1.08)	0.92 (0.80–1.07)
Goats (ref no goats)	0.96 (0.92–1.01)	1.01 (0.86–1.19)	0.90 (0.79–1.03)	0.95 (0.81–1.12)

RRs or betas and 95 % CI were adjusted for age, gender and number of chronic diseases. Bold type indicates statistical significance (*p* < 0.05). Presence of farm animals within 500 m adjusted for the presence of other types of livestock

^a^RR or beta and 95 % CI for an IQR increase in log-transformed exposure. IQR for ln(PM10, g∙y^-1^∙m^-2^) = 10.31, IQR for ln(distance to the nearest farm, m) = 0.82, IQR for ln(distance to GP practice, m) = 1.60, corresponding to a 30,000-fold increase (exp^10.31^), a 2.28-fold increase (exp^0.82^) and a 4.96-fold increase (exp^1.60^) for non-transformed values. Increased RRs for ‘distance to the nearest farm’ indicate that living further away from the nearest farm is associated with an increased odds of disease, i.e. an inverse association with farm exposure

### Association between livestock exposure and self-reported symptoms

The mean number of self-reported symptoms in the previous month was 6.95 (SD: 4.75) for all symptoms and 1.19 (SD: 1.25) for respiratory symptoms. The presence of one or more livestock farms within 500 m of the home address was associated with a reduced reporting of symptoms (RR:0.86; 95 % CI: 0.81–0.92) and respiratory symptoms (RR: 0.80; 95 % CI:0.67–0.96). Distance to general practice was not associated with self-reported symptoms (RR for IQR-range difference in distance being 0.98 (95 % CI: 0.95–1.01) for all symptoms and 0.97 (95 % CI: 0.89–1.06) for respiratory symptoms), and did not confound the association with livestock exposure.

## Discussion

In general, increased livestock exposure was associated with a decreased number of contacts for respiratory conditions and self-reported respiratory symptoms. This difference was not explained by the distance from home to general practice. Thus, distance to general practice did not confound this association. Presence of poultry farms within a 500 meter radius of the home address was associated with more GP contacts in general and specifically contacts for respiratory diagnoses and acute respiratory infections.

Even though our study was conducted in a rural area, the distance from home to the general practice was limited (IQR: 470-2,330 m) in comparison to other studies in for example Australia [7]. Despite the limited distance, we still found a lower contact rate with increased distance to the general practice, indicating a lower accessibility. Future research should focus on the distance at which accessibility to general practice is considered to be reduced.

This study showed that healthcare utilisation is lower with increased livestock exposure. Latent demand indicated by self-reported symptoms is lower as well. It is not easy to give a straightforward explanation for these observations. We may hypothesise that either people living nearby livestock farms are healthier (lower demand) or that they represent a population with a different attitude towards reporting of symptoms and visiting healthcare providers. A previous study using the same data found a lower prevalence of asthma, allergic rhinitis and COPD with higher livestock exposure, indicating better respiratory health in the neighbourhood of livestock farms [10]. Several studies have shown lower risk of respiratory allergies in farm children, which has been attributed to higher exposure to a wide range of microbes [15–17]. In the questionnaire study, patients with lower back pain were also asked to rate their general health using a 5 point Likert scale (bad to very good). The presence of one or more livestock farms within 500 m of the home address was associated with increased general health, although statistically non-significant (multiple ordinal logistic regression, OR: 1.27; 95 % CI: 0.90–1.80). Smit et al. also showed that living within 500 m of at least one livestock farm was associated with a higher education, being raised on a farm and having one or more pets at home [10]. Higher education has been associated with reduced healthcare utilization and better health status [18, 19]. However, correcting associations between livestock exposure and self-reported symptoms for education level did not change associations between livestock exposure and self-reported symptoms (not shown). Furthermore, persons with a respiratory condition attributed to exposure to livestock farms might move to a house with less exposure to livestock farming. More detailed studies should address the relation between health problems, environmental factors, and moving behaviour, by including the history of moving in the analyses.

Previous studies showed that the appreciation of healthcare is higher in persons living in rural areas compared to urban areas, indicating differences in attitude [20]. Although all our study subjects lived in a rural area, people living close to livestock farms may represent a population with a different attitude and could therefore have reported better general health and less complaints. If this is the case, healthcare utilisation could be delayed care with a risk of increased severity of complaints. Future research should focus on the health of specific patient groups, such as patients with chronic respiratory diseases, and especially new cases.

Increased contacts in general practice for respiratory diagnoses and acute respiratory infections with exposure to poultry farms was also shown by Smit et al.. They found an increased incidence of pneumonia in close proximity of poultry farms in the same area in the Netherlands [5]. Information on potential exposures associated with proximity of livestock farms in general is scarce [4]. As a result, it is uncertain which compounds could cause these health effects. Zoonotic viruses and bacteria can circulate in poultry and lead to human exposure and potentially to health effects including pneumonia. Poultry farms emit a wide-diversity of microbes, including viruses, gram-negative and gram-positive bacteria, some of which may be pathogenic [21]. Examples are low and high pathogenic avian flu and Chlamydia psittaci [22, 23]. Gram-negative and gram-positive bacteria have shown to elicit non-infectious inflammatory effects, accompanied by dry cough or dyspnoea [24]. Seedorf et al. showed that respirable endotoxin concentrations was highest for poultry, followed by swine and cattle [25]. Furthermore, emission rates are one factor in the dispersal of compounds from livestock farms. Other factors include wind velocity and direction, and vegetation [4]. As was also concluded in the review by May et al., the compounds responsible for respiratory diseases are not completely understood [24]. More research in needed to unravel the causative factors from these complex environments eliciting respiratory health effects.

### Strengths and limitations

The strengths of our study were the objective assessment at the individual level of the presence of livestock farms around the home address, inclusion of the number of various types of livestock in the proximity of residents, and the analyses of healthcare utilisation through the use of EMR data of general practices. A drawback of using GP records is the limited number of potential confounders available. We only adjusted analyses for gender, age and chronic diseases, but not for socioeconomic status. However, previous research using the same data found no confounding effect of socioeconomic status on the association between livestock exposure and asthma, COPD or allergic rhinitis [10]. Another limitation of our study is the lack of full information on the differences in animal housing systems, such as ventilation and manure handling systems, meteorological influences and practices of land application of manure which may affect local exposure levels. Differential PM10 emission were available depending on the animal type and broad categories of animal housing systems, but we did not take meteorological influences into account. We excluded persons living less than 50 m from a livestock farm, as we were interested in people living close to livestock farms and not people living on farms. This selection might have led to an exclusion of the most affected people. However, analyses including persons living less than 50 m from a livestock farm showed similar estimates. Only lower RRs were found for the associations between poultry and the number of contacts (not shown). Only 531 of the 1519 (35 %) patients with lower back pain returned a completed questionnaire with no missing data. Non-responder analyses showed that participants were more often female, of higher age and were more exposed to livestock. In addition, we have no information about differences in socioeconomic status between participants and non-participants. However, a subsequent study in the same area showed similar negative associations between livestock exposure and the prevalence of respiratory diagnoses in analyses with and without non-responders [26].

## Conclusions

People living in the proximity of livestock farms less often see their GP and also less often report (respiratory) symptoms. Distance from home to the general practice did not confound these associations. This may reflect a different health status (lower demand), or a different attitude towards symptom reporting or healthcare seeking, and may have important implications for epidemiological studies on the proximity to livestock farms and healthcare utilisation. If lower reporting of symptoms and less healthcare utilisation are due to differences in attitude, it may not represent the underlying demand for healthcare. But it could also influence epidemiological results on livestock exposure and health. If people do not report symptoms or do not visit healthcare provider for health problems, this could lead to an underestimation of the effect between livestock exposure and health.

## Appendix

### Appendix 1**.** Fine dust emission estimates.

The license database of the province contains average yearly PM10 dust emission levels (PM10, g per year) for each farm. PM10 emission factors (g per year per animal) have been established by measurements*. Farm emission levels are calculated by summing the products of estimated PM10 emission factors, and the number of allowed animals per stable of all stables on the farm. Weighted dust emissions from all farms within 500 m and 1000 m from the home address were calculated by summing the products of the squared inverse of the distance between a farm and a home address and the farm’s fine dust emission (Σ Distance weighted PM10, g per year per m2).

* Reference: Hofschreuder, P. ,Aarnink, A.J.A., Ogink, N.W.M., Measurement protocol for emissions of fine dust from animal housings, Wageningen : Animal Sciences Group, 2007

### Appendix 2.

**Table 3:** Association of livestock exposures and contacts in general practice adjusted for distance to general practice in 144,984 person years between 2006 and 2009

	All contacts	Contacts for respiratory symptoms	Contacts for respiratory diagnoses	Contacts for acute respiratory infections
	RR (95 % CI)	RR (95 % CI)	RR (95 % CI)	RR (95 % CI)
*Livestock exposures*				
Distance weighted PM10 emission from farms within 500 m^a^	**0.96 (0.94-0.97)**	**0.89 (0.84-0.94)**	**0.93 (0.88-0.98)**	**0.94 (0.89-1.00)**
Distance to the nearest farm^a^	**1.04 (1.03-1.05)**	**1.09 (1.05-1.13)**	**1.07 (1.04-1.10)**	**1.07 (1.03-1.11)**
One or more farms within 500 m (ref no farms)	**0.97 (0.95-0.99)**	**0.92 (0.87-0.97)**	**0.94 (0.90-0.98)**	0.95 (0.90-1.00)
Number of farms within 500 m	**0.99 (0.99-0.99)**	**0.98 (0.96-0.99)**	**0.99 (0.98-0.995)**	0.98 (0.95-1.00)
Presence of farm animals within 500 m				
Cattle (ref no cattle)				
1-69	0.99 (0.97-1.02)	1.00 (0.92-1.08)	0.98 (0.92-1.05)	0.98 (0.91-1.06)
70-299	**0.96 (0.94-0.99)**	0.99 (0.92-1.07)	0.95 (0.90-1.02)	0.96 (0.89-1.04)
300-4,210	**0.96 (0.94-0.98)**	0.95 (0.88-1.04)	0.95 (0.89-1.02)	0.95 (0.87-1.03)
Swine (ref no swine)				
1-649	1.00 (0.97-1.03)	**0.86 (0.79-0.94)**	0.99 (0.92-1.06)	1.01 (0.93-1.10)
650-1,999	0.99 (0.96-1.01)	0.93 (0.85-1.01)	0.95 (0.89-1.02)	0.99 (0.91-1.08)
2,000-32,660	**0.94 (0.91-0.96)**	**0.83 (0.76-0.90)**	**0.89 (0.83-0.96)**	**0.89 (0.82-0.97)**
Poultry (ref no poultry)				
1-13,999	0.99 (0.97-1.02)	0.91 (0.82-1.01)	1.03 (0.95-1.13)	1.00 (0.90-1.11)
14,000-401,250	**1.05 (1.02-1.08)**	1.08 (0.98-1.19)	**1.09 (1.00-1.18)**	**1.17 (1.06-1.30)**
Sheep (ref no sheep)				
1-49	0.99 (0.96-1.02)	1.02 (0.92-1.13)	1.01 (0.93-1.10)	0.98 (0.88-1.09)
50-1400	1.00 (0.97-1.04)	1.04 (0.93-1.17)	1.03 (0.93-1.13)	1.00 (0.89-1.12)
Minks (ref no minks)	**0.95 (0.91-0.99)**	0.93 (0.81-1.07)	0.97 (0.87-1.09)	0.94 (0.81-1.08)
Goats (ref no goats)	0.96 (0.91-1.01)	1.01 (0.86-1.19)	0.90 (0.79-1.03)	0.95 (0.80-1.12)

RRs or betas and 95 % CI were adjusted for age, gender and number of chronic diseases. Bold type indicates statistical significance (*p*<0.05). Presence of farm animals within 500 m adjusted for the presence of other types of livestock. ^a^RR or beta and 95 % CI for an IQR increase in log-transformed exposure. IQR for ln(PM10, g∙y^-1^∙m^-2^) = 10.31, IQR for ln(distance to the nearest farm, m) = 0.82, IQR for ln(distance to GP practice, m)=1.60, corresponding to a 30,000-fold increase (exp^10.31^), a 2.28-fold increase (exp^0.82^) and a 4.96-fold increase (exp^1.60^) for non-transformed values. Increased RRs for ‘distance to the nearest farm’ indicate that living further away from the nearest farm is associated with an increased odds of disease, i.e. an inverse association with farm exposure.
